# Technology and College Student Mental Health: Challenges and Opportunities

**DOI:** 10.3389/fpsyt.2019.00246

**Published:** 2019-04-15

**Authors:** Emily G. Lattie, Sarah Ketchen Lipson, Daniel Eisenberg

**Affiliations:** ^1^Center for Behavioral Intervention Technologies, Department of Medical Social Sciences, Northwestern University, Chicago, IL, United States; ^2^Boston University School of Public Health, Department of Health Law Policy and Management, Boston University, Boston, MA, United States; ^3^University of Michigan School of Public Health, Department of Health Management and Policy, and Population Studies Center, Institute for Social Research, University of Michigan, Ann Arbor, MI, United States

**Keywords:** mental health, college students, digital mental health, health services, smartphones

## Abstract

In recent years, there has been an increase in symptoms of depression, anxiety, eating disorders, and other mental illnesses in college student populations. Simultaneously, there has been a steady rise in the demand for counseling services. These trends have been viewed by some as a mental health crisis requiring prompt investigation and the generation of potential solutions to serve the needs of students. Subsequently, several studies linked the observed rise in symptoms with the ubiquitous rise in use of personal computing technologies, including social media, and have suggested that time spent on these types of technologies is directly correlated with poor mental health. While use of personal computing technologies has dramatically shifted the landscape in which college students connect with one another and appears to have some detriments to mental health, the same technologies also offer a number of opportunities for the enhancement of mental health and the treatment of mental illness. Here, we describe the challenges and opportunities for college student mental health afforded by personal computing technologies. We highlight opportunities for new research in this area and possibilities for individuals and organizations to engage with these technologies in a more helpful and wellness-promoting manner.

The college years represent an important period with regard to mental health and health behaviors. Nearly 70% of Americans enroll in college immediately following high school (1), and roughly three-quarters of lifetime cases of psychiatric disorders begin by age 24 (2). In recent years, there has been an increase in reported symptoms of mental illnesses in college student populations. A large, epidemiological study recently demonstrated that mental health diagnoses have risen from 22% to 36% among college student respondents over the last 10 years (3). In a survey of college counseling center directors, more than 95% reported that the number of students with significant psychological problems was a growing concern on their campus (4). Across the country, more and more college students appear to be suffering.

The clinical importance of this phase of life is compounded by the fact that college students report numerous barriers to mental health treatment. Many students do not recognize a treatment need, believing that clinically significant symptoms of depression and anxiety are typical of college life (5). Students recognizing a need for treatment often report difficulties accessing care, perceive available care as inconvenient, and are skeptical about the efficacy of care (6, 7). However, over the last 10 years as mental health diagnoses have risen and stigma has decreased, there has been a dramatic rise in students seeking mental health services (from 19% to 34%) (3). Many campus communities find themselves unable to keep up with this demand (8).

Some people have viewed the observed increases in symptoms of mental illness and demand for services as a “campus mental health crisis” (8, 9). Many have begun to speculate on causes of this presumed crisis. A portion of these increases might be due to mental health support offered prior to college, which has assisted students in getting to college (10), and due to reductions in mental health stigma, which may result in students being more willing to disclose and seek assistance for mental health difficulties (3, 11).

As personal computing technologies, such as smartphones with their easy accessibility to social media outlets, have become increasingly ubiquitous, they have increasingly been the subject of examination as a potential cause of poor mental health or as a trigger of this mental health crisis (12, 13). The ubiquity of smartphones has presented a shift in how people communicate. According to the latest Pew Research Center study in early 2018, approximately 91% of Americans aged 18–29 now own smartphones (14). A cultural norm of young people being constantly connected to the Internet has been established, with traditional college-aged Americans (ages 18–24) estimating that they look at their phones more than 80 times per day (15).

There are a number of challenges and opportunities for college student mental health afforded by personal computing technologies such as smartphones. We are living in a time of unprecedented social connection and access to educational resources. Some may argue that we are simultaneously living in a time of unprecedented awareness of social exclusion and information overload. This has been made apparent by the emergence of “fear of missing out” or FoMO, which was first discussed by a marketing strategist (16), and since has become the subject of several empirical investigations on mental health and social media use (17–19).

Given that the rise in use of personal computing technologies and social media has occurred concurrently with a rise in young adults reporting mental health symptoms, one may be tempted to conclude that these types of tools are unhealthy. Some have proposed that the high level of connectivity associated with these tools, combined with a decrease in the quality and quantity of face-to-face social interactions, is a large contributor to the observed rise in distress among youth (20, 21). While several studies have found that time spent on social media and use of multiple social media platforms is associated with poor mental health (22–24), a more recent, particularly rigorous examination of three large datasets found that while the association between personal computing technology use and adolescent well-being was negative, it explained at most 0.4% of the variation in well-being (25). Further, these studies have been correlational and thus cannot assert causality. Rather than thinking of social media as inevitably causing poor mental health, it may be more useful to distinguish between healthy and unhealthy use of social media. For example, some evidence suggests that Facebook use is only harmful to mental health when it is passive viewing of other people’s posts (26) as opposed to more active engagement in social connections (see **Table 1** for a review of these findings).

**Table 1:** Summary of findings.

Proposed negative effects of personal computing technology use on mental health	Proposed positive effects of personal computing technology use on mental health	Existing technology-enabled interventions
Fear of missing out Hyper-connectivity with peers Peer comparison Decreased face-to-face social interactions Impairment of social skill development Decreased inhibition of anti-social behavior	Active engagement with peers Expanded social networks Venues for personal disclosures Peers can serve as “gatekeepers” Access to mental health intervention programs	Online support groups and message boards Module-based web interventions (e.g., MoodGYM, Beating the Blues) Skill-building apps (e.g., Headspace, Pacifica) for resilience, coping skills, mindfulness

It is clear that the rise in personal computing technology usage has dramatically shifted the landscape in which college students connect with one another. While misuse appears to have some detriments to mental health, the same technologies offer a number of opportunities for the enhancement of mental health and the treatment of mental illness. As with nearly every behavior, moderation is key. It would be irresponsible to conclude that smartphone usage and social media are inherently bad, as they serve as pathways to connect individuals with their existing social support networks, and as pathways for individuals to build new social support networks. Indeed, social media platforms, such as Facebook and Instagram, are increasingly seen as venues for personal disclosures and for establishing and maintaining social connections (27–29).

Further, continuous access to personal computing *via* one’s smartphone brings with it a host of opportunities for mental health intervention programs. Technology-enabled mental health services, including those delivered online and *via* apps, offer the possibility to expand treatment options and reduce barriers to mental health services. Among multiple subpopulations (from children to older adults) and for many presenting problems (including depression and anxiety), technology-enabled mental health services have demonstrated efficacy (30, 31).

A body of literature on technology-enabled mental health interventions specifically for college students demonstrates that website- and computer-delivered programs can be effective for improving depression, anxiety, and well-being outcomes (32). This is not surprising, as students are living an increasing portion of their lives online, and thus, it may be wise to meet them where they are. To date, most research on technology-enabled mental health programs for college students have been on programs delivered *via* website and based on cognitive–behavioral therapy principles, such as MoodGYM (33–35) and Beating the Blues (36, 37). As college students spend an increasing portion of their online lives connected *via* smartphones rather than desktop or laptop computers (38), technology-enabled mental health programs need to be designed for smartphone accessibility, and the use of native apps and web apps should be considered. Smartphone apps, when well-designed, are increasingly seen as effective for delivering mental health interventions (39). Early trials of mobile programs designed specifically for college students are promising and have demonstrated improvements in users’ stress, depression, anxiety, and productivity (40, 41).

While there appears to be significant potential for both web-based and app-based programs, the majority of technology-enabled mental health programs for college students have not been examined outside the context of tightly controlled research studies of interested (i.e., self-selecting) student participants. There appears to be a significant research-to-practice gap such that technology-enabled mental health intervention programs are largely underutilized on college campuses (42, 43) and few research studies have focused on the implementation of such programs within college counseling centers (37, 44).

Administrators and health professionals on college campuses have already shown some degree of openness to providing technology-enabled mental health resources. Perhaps most notably, technology-enabled mental health screening programs have been well received (45, 46) and have been implemented on campuses across the country. Technology-enabled mental health screening programs have typically been tools available on campus websites, in which students can complete standardized screening measures for common mental health and behavioral problems (e.g., depression, anxiety, disordered eating, and alcohol misuse) and receive feedback regarding the results of the assessment completed. These programs have shown promising evidence of effectiveness as a way to identify students in distress and link them to services (47, 48). Where the research-to-practice gap exists prominently is for technology-enabled mental health services, in which students could access self-guided or coached therapeutic programs *via* the web or apps. While students could theoretically access these types of programs without involvement from their campus community, counseling centers are unlikely to see reductions in their ever-increasing workload without coordinated efforts to educate students on, and direct them to, technology-enabled programs. Because college counseling centers across the United States are frequently understaffed, have limited budgets, and operate on waitlists for much of the year (8), it may be in their best interest to support the incorporation of technology-enabled mental health programs into routine practice, as a supplement to their core services.

The integration of technology-enabled services into routine practice will not be without challenges to clinical providers, including challenges related to ethics, accountability, duty of care, and privacy. Provided that technology-enabled mental health services are not part of standard practice in most settings, campus mental health providers may not have proper education on the ethical use of these tools. While there are no nationally sanctioned guidelines, there remains a need for continuing education on the unique set of ethical considerations that go into use of these tools (49).

To address concerns about accountability and duty of care, we recommend that providers develop written consent for treatment forms that highlight the appropriate uses of and limitations of a particular technology-enabled service. Specifically, students who are consenting to using technology-enabled mental health services should be made aware of how their data (including, but not limited to, anything they type into a program) will be used and monitored, and how they are to contact and communicate with clinical staff. If students are able to send messages to a clinical provider, or if a clinical provider has access to information that students type into a program, there must be clearly agreed-upon expectations regarding how frequently this information will be reviewed, and about what student users are to do in the event of a mental health emergency (e.g., present to the emergency room or call the campus crisis number, rather than messaging the mental health provider). The process of developing these consent forms, and having them approved by the appropriate administrators within a campus health system, is a valuable opportunity to clarify, or perhaps establish, university policies on technology-enabled services.

Privacy is also an important concern that deserves to be thoroughly addressed. Some clinicians may shy away from using technology-enabled tools for fear of poor privacy protections. Indeed, recent studies have found that the majority of commercially available mental health apps had insufficient privacy policies (50) and that these privacy policies are frequently incomprehensible (51). Campus mental health providers may find it useful to collectively generate a list or library of technology-enabled tools that meet their agreed-upon standards for privacy. In our interactions with campus mental health providers, we repeatedly hear requests for more education regarding which digital programs are most helpful for particular issues and guidance around privacy standards. Fortunately, user-friendly resources (e.g., PsyberGuide, an online mental health app guide available at www.psyberguide.org) are continually being developed and refined and are freely available to the public.

To move the field forward and to begin to offer more resources to alleviate student suffering, there must be better integration between services available on campus and services available in students’ digital lives. At a basic level, there continues to be a significant need for research to identify best practices for supporting students to use personal communication technologies in a prosocial manner. While college counseling center staff around the country may be guiding students toward more healthful use of these technologies, we are not aware of any research on effective strategies for intervening with students at this level. The development of protocols for supporting students in making healthier choices in their digital lives (e.g., maximizing appropriate social support seeking, minimizing unhelpful social comparisons) and in their physical lives (e.g., exercising, spending time with friends in face-to-face settings) could be a first step in guiding students toward improved mental health. These types of protocols may be modeled off of a harm-reduction approach consistent with what Veissière and Stendel recently proposed (52), as we recognize the normal, healthy need for social connection that these technologies offer and would be very hesitant to recommend anything that would require full abstinence from these technologies.

At a more advanced level, we need to identify best practices in designing technology-enabled mental health programs that students will want to use, and best practices for implementing these types of programs on campuses.

User engagement is a major challenge for technology-enabled mental health services, even more so than for traditional in-person services. However, college communities have key advantages: a population with high comfort using the technologies, and also a range of in-person points of connection that could enhance engagement with the technologies. Thus, the key question is how to leverage those advantages most effectively. As campus professionals determine programs that are appropriate for their needs, they may implement these programs in a variety of ways, including blended models of therapy, with both in-person and digital components (for both individuals and groups), and stepped care approaches, in which students may first receive digital interventions and those who do not respond are stepped up to traditional in-person care. Additionally, colleges may begin integrating mental health resources into the digital services that students are already using regularly, such as posting content about digital mental health tools directly onto course software programs.

If current trends continue (i.e., rising prevalence of symptoms, increasing rates of help-seeking, particularly of campus counseling center resources), demand will continue to outpace supply. The stakes for this are very high, given the epidemiological vulnerability of the college years and the still largely missed opportunity for early intervention, prevention, and treatment (i.e., all levels of public health programming). Personal communication technologies and the technology-enabled services that can be made available through them are undeniable parts of this landscape, and much of the potential remains untapped.

## Author Contributions

EL, SL, and DE contributed to the conception of the manuscript. EL wrote the first draft. SL and DE wrote sections of the manuscript. All authors contributed to manuscript revision and read and approved the submitted version.

## Funding

EL is supported by a research grant (K08 MH112878) from the National Institute of Mental Health.

## Conflict of Interest Statement

EL has accepted consulting fees from Actualize Therapy.

The remaining authors declare that the research was conducted in the absence of any commercial or financial relationships that could be construed as a potential conflict of interest.
